# *In vitro* propagation and assessment of genetic stability in date palm as affected by chitosan and thidiazuron combinations

**DOI:** 10.1186/s43141-022-00447-9

**Published:** 2022-12-14

**Authors:** Ahmed Madi Waheed Al-Mayahi

**Affiliations:** Date Palm Research Centre, University of Basrah, Basrah, Iraq

**Keywords:** Micropropagation, Culture media, Multiple shoots, RAPD-PCR marker

## Abstract

**Background:**

Mass propagation of date palm has attracted the interest of commercial producers. However, this technique still faces many obstacles that hinder production. This study investigated the effect of chitosan (CHT) at various concentrations for the possibility to apply it in combination with *thidiazuron* (TDZ) on the growth and development of tissue cultures of Barhee cultivar.

**Results:**

The results showed that CHT and TDZ on in vitro proliferation of Barhee date palm cultivar were significant. The highest response rate and the number of shoots per jar were found in MS media supplemented with 15 mgL^–1^ CHT and 0.5 mgL^–1^ TDZ combination. Furthermore, we found that the combined application between 20 mg L^−1^ CHT+ 1.0 mg L^−1^ TDZ resulted in the highest shoots content of endogenous IAA, compared with other treatments. At the same time, the data revealed that the maximum cytokinins (CKs) content of shoots occurred in a medium supplemented with 15 mg L^−1^ CHT and 0.5 mg L^−1^ TDZ. The genetic stability of the discussed micropropagation protocol was confirmed in this study by DNA-based technique RAPD (random amplified polymorphic DNA). The results may indicate that the micropropagation protocol developed in this research paper was appropriate and applicable for producing genetically stable date palm cv Barhee plants.

**Conclusion:**

Applying the strategy of culture treatment with (CHT) and (TDZ) can be valuable for improving the propagation of date palm cv Barhee in vitro.

## Background

Date palm is a monocotyledon plant from the Arecaceae family. The exact origin of the date palm (*Phoenix dactylifera* L.) is not known precisely. However, according to Mesopotamian antiquities, this plant likely originated in southern Iraq at least 6000 years ago [1]. The high nutritional composition, profitability, and environmental advantages make the date palm a major role in food security and job creation in various countries [2, 3].

The vegetative propagation of the date palm is traditionally achieved by offshoots. This type of offshoots multiplication has limitations such as a slow propagation rate making them less effective in establishing new plantations, the transmission of disease-causing pathogens and insects, and the production of a limited number of offshoots for a certain period through the juvenile phase [4–6]. In vitro culture methods have attracted great interest in overcoming these limitations and producing pathogen-free plants [7].

The positive effect of CHT on in vitro propagation has been proved in some plant species [8–10]. CHT appears as a potential agent used to enhance the plant’s defence mechanism, as a growth stimulator, as an antimicrobial agent; besides this, CHT is used as a carrier to improve nutrient delivery [11]. Cytokinin (CKs) promotes cell division, and stimulates lateral shoot growth [12]. Some commonly used CKs in plants micropropagation include Thidiazuron (TDZ) 1-phenyl-3 (1,2,3-thiadiazol-5-yl) urea. TDZ is the cytokinin-like substance that promotes micropropagation of a wide array of woody species, including date palm, because of its tremendous ability to stimulate shoots proliferation [13, 14]. TDZ is also a powerful regulator of plant propagation in vitro and subsequent growth [15]. Many reports show that the use of TDZ leads to a better shoot proliferation capacity than other CKs [16, 17]. If date palm micropropagation is successful, an evaluation to determine the genetic stability of the cultures would need to be established before a large-scale tissue culture method could be developed for commercial use. Although it may present a severe problem for commercial micropropagation, molecular markers can efficiently verify clonal variations. These variations during tissue culture depend largely on many factors, among them the media composition. Plant tissue culture is particularly suitable for evaluating the compounds and plant growth regulator’s effects on plants that multiply and differentiate slowly, such as the date palm. However, there is no study examining the effect of CHT and TDZ on the in vitro propagation of date palms and related gene expression. Hence, this study investigated the effect of CHT at various concentrations for the possibility to apply it in combination with TDZ on the growth and multiplication in vitro of date palm buds, some physicochemical parameters, and genetic stability; RAPD indicators were used to determine the genetic stability of in vitro multiplied materials to determine the protocol effectiveness.

## Methods

Experiments were conducted in the Department of Date Palm Propagation, Date Palm Research Center, Basrah University, Basrah-Iraq.

**Fig. 1:**
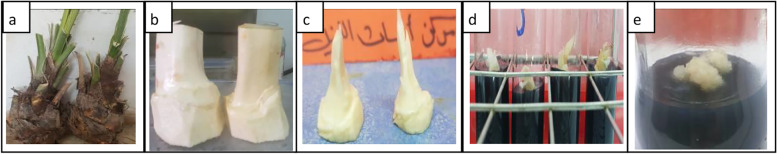
Induction of callus of date palm (*P. dactylifera* L. cv. Barhee) from apical buds of an offshoot: **a** offshoots, **b** trimmed offshoots, **c** apical buds, **d** quarters of apical buds on MS medium, **e** callus formation (× 0.8)

Young offshoots (3 years old) of date palm cv. Barhee was detached from the mother palm (Fig. 1a). Outer leaves and fibrous tissues at their bases were removed gradually until exposure to the shoot tip zone (Fig. 1b, c). Sheathing leaf base enclosing the very young leaves of the heart of the offshoot was left in place to protect it from disinfection solutions. Four apical buds were used in the experiment; each apical bud was sectioned longitudinally into four sections. To callus induction, quarters of the apical buds were transferred to MS basal medium [18], supplemented with 3 mg L^−1^ 6-(dimethylallyl amino) purine (2iP), 30 mg L^−1^ naphthalene acetic acid (NAA), 2.0 g L^−1^ activated charcoal and solidified with Agar-Agar at 7.0 g L^−1^. The cultures were transferred to fresh media, with the same composition every 6 weeks until the callus had initiated (Fig. 1d, e). All cultures were incubated in a culture room under darkness at 27 ± 2 °C for 180 days to initiate callus. The percentage of callus formation reached 62.5%. For callus propagation; it was transferred and grown in jars containing 25 ml of the MS medium supplemented as mentioned above, except NAA at 6 mg L^−1^ and 2iP at 2 mg L^−1^, with reducing the activated charcoal (AC) concentration to 0.5 mg L^−1^ for use in subsequent experimental techniques experiments, where it lasted for 10 weeks. For differentiation and multiplication, the yellow compact calluses on growth media was divided and subcultured on differentiation, and multiplication media. To study the effects of CHT and TDZ, supplementation of these compounds at various concentrations in the culture media was assessed. MS medium was modified at five concentrations of CHT (0.0, 5, 10, 15, and 20 mg L^−1^) alone or in combination with TDZ at three concentrations (0.0, 0.5, and 1.0 mg L^−1^). In order to obtain the best nutritional composition capable of stimulating shoot organogenesis and changes in phytochemicals. Treatments were consisted of 15 media, as shown in Table 1. All cultures were kept in the incubator at a temperature of 25 ± 2 °C. A photoperiod (2000 lux) of 16 h using cool white fluorescent tubes were also provided. The medium pH was adjusted to 5.7 before autoclaving. Subculturing was carried out in a fresh medium every 5 weeks for up to 3 months. The results of the experiments regarding the effect of MS medium on the percentage of shoot regeneration and shoot number per jar were evaluated 12 weeks after the inoculation of callus on the media. There were twelve replicates (jars) of each treatment.

**Table 1:** Treatments that applied in this study

No.	Treatments (mgL^−1^)	No.	Treatments (mgL^−1^)
**1**	0.0 CHT + 0.0 TDZ	**9**	10 CHT + 1.0 TDZ
**2**	0.0 CHT + 0.5 TDZ	**10**	15 CHT + 0.0 TDZ
**3**	0.0 CHT + 1.0 TDZ	**11**	15 CHT + 0.5 TDZ
**4**	5 CHT + 0.0 TDZ	**12**	15 CHT + 1.0 TDZ
**5**	5 CHT + 0.5 TDZ	**13**	20 CHT + 0.0 TDZ
**6**	5 CHT + 1.0TDZ	**14**	20 CHT + 0.5 TDZ
**7**	10 CHT + 0.0 TDZ	**15**	20 CHT + 1.0 TDZ
**8**	10 CHT + 0.5 TDZ		

### Extraction and measurement of auxin (IAA)

Auxins were extracted and quantified according to [19]. Five grams of leaves after various treatments with CHT and TDZ were homogenized using 80% methanol. The extract has been filtered through the Whatman filter paper (no. 1) and evaporated at 4 °C in dark conditions under a vacuum. The supernatant was dried in a vacuum, withdrawn by 0.1 M phosphate potassium (pH 8.1). Eluate was obtained using 1 N hydrochloric acid (HCI) and by partitioning (4×) with diethyl ether, in dryness, in water with a pH set to 2.5. The injection in reversed HPLC, C18 column, in the isocratic elution mode by the concentrate, determined phytohormones using a portable acetone step (26:74) with 30 mM of phosphoric acids. A UV detector (2996PDA detector) with 280 nm was passed through the column eluants, and auxins were detected and quantified. Standard auxins were used as the source (IAA).

### Determination of cytokinins (CKs)-like substances

The CKs-like substances in the shoot tissue were extracted and quantified [20]. Spectrophotometer UV-Visible Shimadzu determined the shoot samples’ CK-like substances at the wavelength of 265 nm. The values content of the endogenous CK was calculated using the linearized standard curve in which benzyl adenine was used, and the results were expressed in micrograms. The values were corrected, and the endogenous CKs content was calculated using the linearized standard curve in which benzyl adenine was used, and the results were expressed in micrograms.

### Genetic stability among regenerated date palm plantlets

In order to study the genetic similarities, several regenerated plantlets were analyzed at the molecular levels using RAPD analysis.

### RAPD analysis

Total genomic deoxyribonucleic acid (DNA) was isolated from regenerated date palm plantlets using the CTAB method described in [21]. Polymerase chain reaction (PCR) reactions were conducted using a set of four arbitrary 4-mer primers (Operon Technology, Inc., Alameda, CA, USA). These primers and their sequences are presented in Table 2.

**Table 2:** The RAPD primers and their sequences used for the genetic fidelity evaluation

Primers	Sequences
**OPA-02**	**TGCCGAGCTG**
**OPB-05**	**TGCGCCCTTC**
**OPE-15**	**ACGCACAACC**
**OpO-07**	**CAGCACTGAC**

### The PCR mixture

The reaction mixture (20 μl) contained 10 ng DNA, 200 μM deoxynucleotide triphosphates (dNTPs), 1 μM primer, 0.5 units of Red Hot Taq polymerase (AB-gene Housse, UK) and 10-X Taq polymerase buffer (AB-gene Housse, UK). For DNA amplification, a Perkin Elmer thermal cycler (2720) programmed as follows: Denaturing: 95 °C for 5 min 94 °C for 0.45 min. Then, annealing (35 cycles) 35 °C for 1 min. This is followed by 72 °C for 1 min and 30 s and finally Extension: at 72 °C for 7 min [22]. The amplification products were separated in 1% (w/v) agarose gel in 1× Tris/Borate/Ethylenediaminetetraacetic_acid (TBE) buffer and visualized by staining with ethidum bromide. The reproducibility of DNA profiles was determined by replicating all RAPD reactions at least three times using DNA markers. The primers were evaluated from wise pair comparison for the proportion of shared bands amplified [23]. The similarity coefficient was calculated by using the statistical software package STATISTICA-SPSS (Stat Soft Inc.).

### Statistical data analysis

In all experiments, each treatment consisted of 12 replicates. Data were statistically analyzed by analysis of variance (ANOVA). The least significant difference (LSD) method was used to test the difference between treatments, and *p* ≤ 0.05 was considered statistically significant. Statistical analyses were performed with SPSS packet software.

## Results

### Effect of CHT and TDZ on shoot induction

**Fig. 2:**
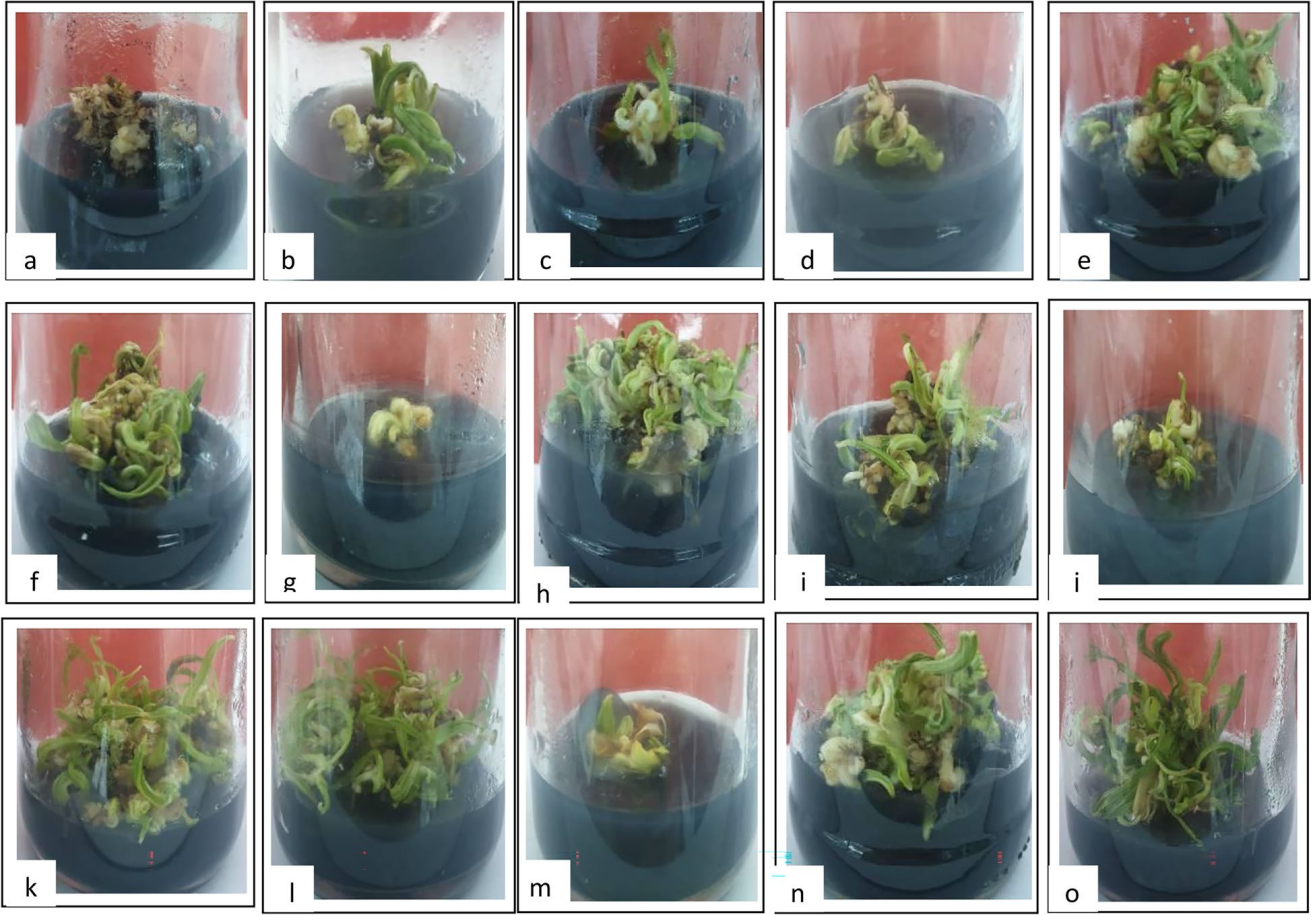
Initiation of multiple shoot formation after 12 weeks of culture on MS media supplemented with chitosan (CHT) alone or in combination with thidiazuron (TDZ): mgL^−1^: **a** control, **b** 0 CHT + 0.5 TDZ, **c** 0 CHT + 1.0 TDZ, **d** 5 CHT + 0.0 TDZ, **e** 5 CHT + 0.5 BA, **f** 5 CHT + 1.0 TDZ, **g** 10 CHT + 0.0 TDZ, **h** 10 CHT + 0.5 TDZ, **i** 10 CHT + 1.0 TDZ, **j** 15 CHT + 0.0 TDZ, **k** 15 CHT + 0.5 TDZ, **l** 15 CHT + 1.0 TDZ, **m** 20 CHT + 0.0 TDZ, **n** 20 CHT + 0.5 TDZ, **o** 20 CHT+ 1.0 TDZ. (× 0.8)

According to the results obtained, the combination between CHT and TDZ application had the highest response percentage and number of shoot compared with either individual application or control treatment (Fig. 2e, f, h, i, k, l, n, and o) after 12 weeks from culture. The response rate and the number of developing shoots varied among the different treatments (Table 2). The highest response rate and the number of developing shoots, 75% and 16.11 respectively, were obtained on the medium supplemented with 15 mg L^−1^ CHT and 0.5 mg L^−1^ TDZ (Fig. 2k).

### Endogenous hormones levels

#### Endogenous IAA levels

**Fig. 3:**
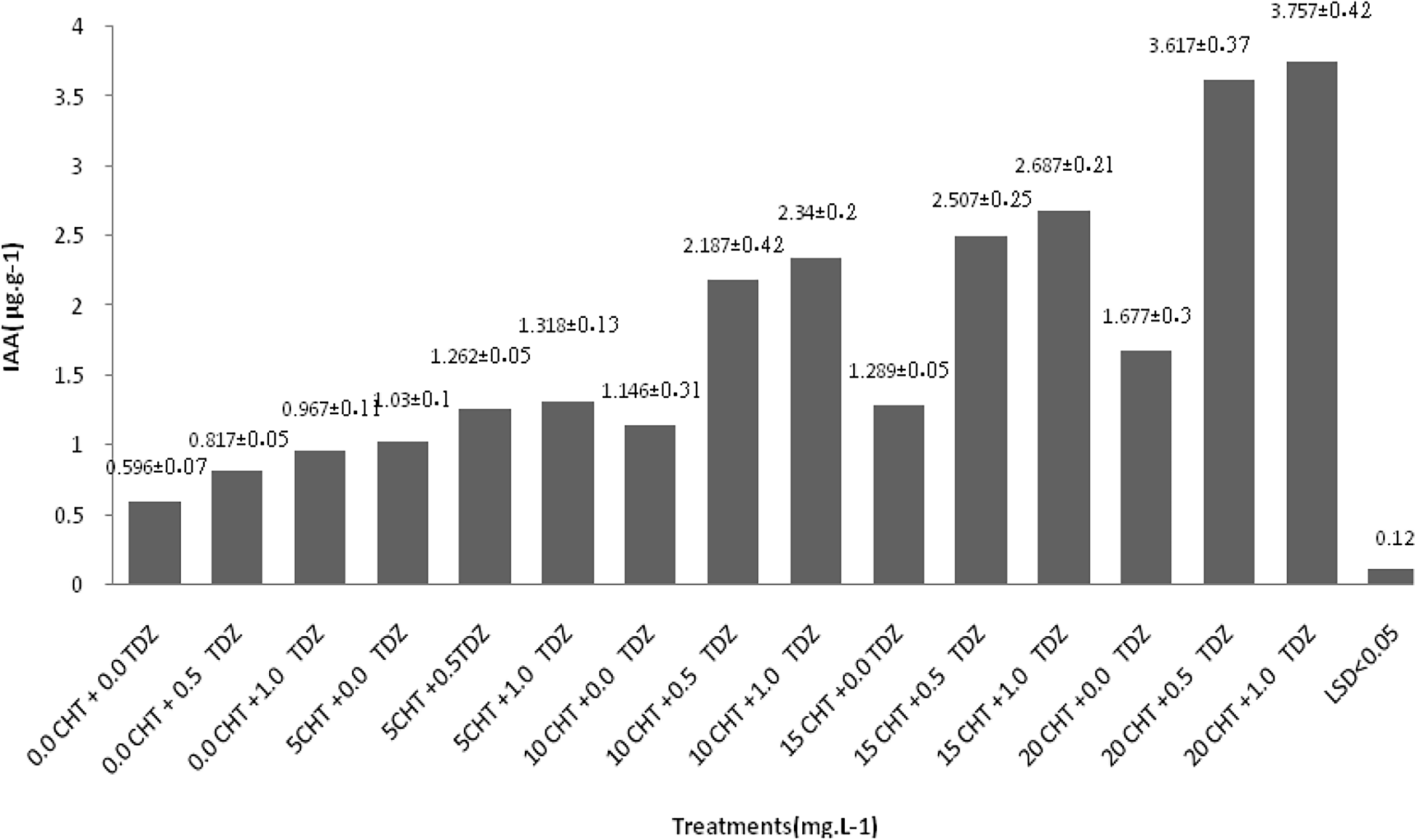
Effect of chitosan (CHT) and thidiazuron (TDZ) on endogenous indole acetic acid (IAA) content. ± Standard error (n15)

Figure 3 shows the effect of CHT and TDZ on endogenous IAA content under in vitro conditions. Among all treatments, CHT and TDZ were applied. The highest IAA content (3.757 μg kg^−1^) in the shoots was obtained in the MS medium supplemented with 20 mg L^−1^ CHT + 1.0 mg L^−1^ TDZ. On the other hand, the lowest contents were recorded in shoots grown in the control medium.

#### Endogenous cytokinin (CK) levels

**Fig. 4:**
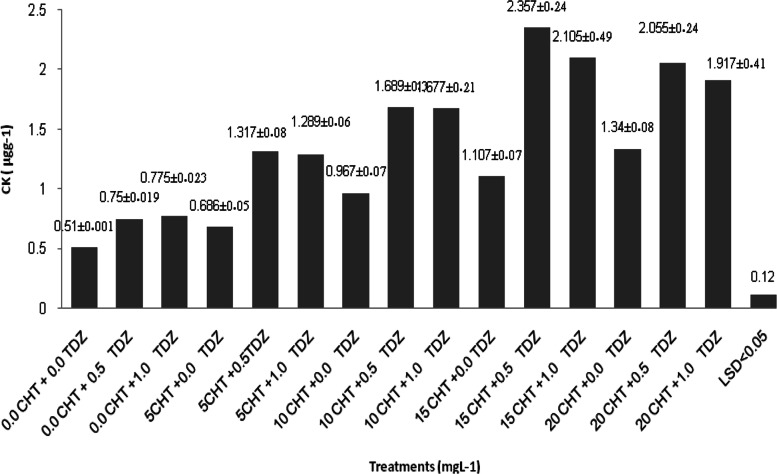
Effect of chitosan (CHT) and thidiazuron (TDZ) on endogenous cytokinin (CK) content. ± Standard error (n15)

Figure 4 shows the effect of CHT and TDZ on endogenous CKs concentrations. Thus, increasing the CHT concentration of the medium from 0.0 to 20 mg L^−1^ resulted in a proportionally increasing content of endogenous CK of tissues, as observed when TDZ concentration was increased from 0.0 to 1.0 mg L^−1^. Furthermore, in regeneration media with 15 mg L^−1^ CHT + 0.5 mg L^−1^ TDZ , the highest CKs content was recorded (2.327 μg kg^−1^,) which was significantly different than what was reported at the shoots grown in the other media (*p* < 0.05). The lowest contents were recorded in shoots grown in the control medium (with no additives).

#### RAPD-based genetic relationships

**Fig. 5:**
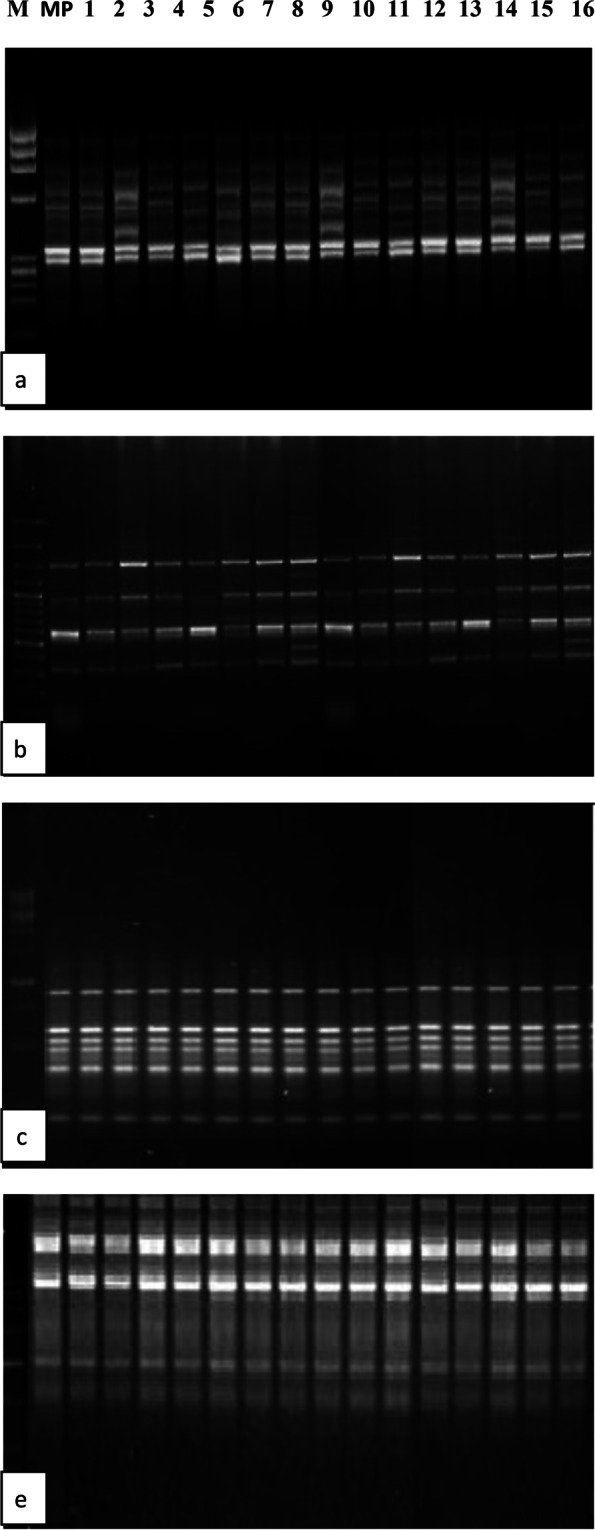
**a** OPB-05, **b** OPE-08, **c** OPE-15, and **d** OpO-07). RAPD pattern of regenerated plants of *Phoenix dactylifera* L. on MS medium supplemented with chitosan (CHT) alone or in combination with thidiazuron (TDZ): mgL^−1^:(M) size marker (1) (1) MP: mother plant, (2) Control (3) 0 CHT + 0.5 TDZ (4) 0 CHT + 1.0 TDZ (5) 5 CHT + 0.0 TDZ (6) 5 CHT + 0.5 BA (7) 5 CHT + 1.0 TDZ (8) 10 CHT + 0.0 TDZ (9) 10 CHT + 0.5 TDZ (10) 10 CHT + 1.0 TDZ (11) 15 CHT + 0.0 TDZ (12) 15 CHT + 0.5 TDZ (13) 15 CHT + 1.0 TDZ (14) 20 CHT +0.0 TDZ (15) 20 CHT + 0.5 TDZ(16) 20 CHT + 1.0 TDZ

In the present study, we regenerated plants from callus tissues under the influence of different CHT and TDZ. Since morphological evaluation is unreliable in characterizing variability among the tissue culture-derived plantlets with the mother plant, it becomes necessary to check the genetic stability of these regenerated plants. Four operon primers were used (OPB-05, OPE-08, OpO-07, and OPE-15). All of them have given good amplification with scorable DNA bands. The results also revealed that the regenerated plants did not have any polymorphism with the mother plant (Fig. 5a-d). DNA bands produced by amplification with RAPD markers were monomorphic across the mother plant and micropropagated plantlets (Fig. 5). The genetic profiles obtained in RAPD analysis suggest no genetic changes between the tissue culture-derived plantlets treated with CHT and TDZ with the mother plant. This suggests that the in vitro conditions in this study provide relatively genetic stability of date palm cv Barhee on regeneration in vitro, which confirms the true-to-type and genetically stable nature of those plants. The fidelity of the micropropagation protocol to produce true–to–type date palm plants indicate that the use of CHT and TDZ during in vitro propagation phases showed a similar banding pattern in vitro regenerated and mother plant in RAPD profiles of this date palm cv. Barhee.

## Discussion

In vitro propagation of date palm depends on the appropriate selection of explants and hormones [24, 25]. Due to the preferred properties of CHT, it can be used as a growth stimulator in vitro of date palm. To determine a suitable medium for an efficient in vitro propagation of date palm cv. Barhee, callus tissues were cultured on MS media supplemented with different CHT and TDZ combinations. MS media supplemented with CHT in combination with TDZ gave the best response on most growth criteria studied as compared with no additives (control treatment) or with their individual application. The highest response rate and the number of shoots per jar were achieved in cultures incubated on MS medium supplemented with 15 mg L^−1^ CHT + 0.5 mgL^−1^ TDZ (Table 1). The interesting property of CHT suggests its potential as a growth regulator for the micropropagation of date palm. Using MS media with different chitosan concentrations may enhance the response rate and the number of shoots produced per jar. CHT can be used as a plant growth enhancer. Perhaps CHT stimulates a signal for the synthesis of plant hormones such as gibberellins. In addition, CHT may promote growth and development through some signaling pathways related to auxin biosynthesis by the tryptophan-independent pathway [26, 27] who indicated that the use of CHT stimulated the accumulation of auxin. CHT, a natural alternative to commonly used synthetic plant growth regulators, in vitro effects of chitosans have been mainly investigated based on morphometric parameters such as the number of shoots, roots, and leaves [28, 29]. Chitosan treatment has been shown to improve the growth of in vitro-cultured plantlets of bananas and enhance immunity against various plant pathogens [30]. This study confirmed that CHT could act as a plant growth stimulator for date palm cv Barhee micropropagation. These results agree with those of [31], who reported that CHT was a stimulator for enhancing the growth of safflower and sunflower plants, and with [32] in *Petunia atkinsiana* D. don.). Thus, suggested that CHT was able to enhance the growth of date palm ‘Barhee’ was reported in this paper was in accordance to many many other researches in many plants [8, 9, 30, 33]. On the other hand, TDZ is necessary for date palm propagation in in vitro. The use of TDZ in culture media enhanced the growth traits. This is probably due to the difference in endogenous levels of growth regulators, where TDZ plays a vital role in stimulating endogenous cytokinin biosynthesis or altering cytokinin metabolism. An examination of the different media and plant growth regulators combinations that have been utilized for tissue culture of date palm plants reveals that shoot regeneration requires the presence of both auxins (Aux) and cytokinins (CKs) [34]. This was consistent with findings on applications of TDZ in shoot regeneration and the synergistic effect of TDZ with auxins on the proliferation higher number of shoots [16]. Auxins play a vital role in the differentiation of cell aggregates which is a prerequisite for regeneration. Additionally, auxin is one of the plant hormones necessary for the growth and development of in vitro organs [35]. Aux (e.g., IAA) is produced in response to physiological or metabolic changes [36]. We found that increasing CHT leads to the accumulation of IAA and CKs related to plant growth. In addition, there is evidence that CHT can be used as an alternative to commonly used growth factors such as Aux or CKs [37, 38] due to its stimulating effects [39]. In our study, tissue exposure to increased chitosan levels leads to the accumulation of IAA in buds. This hormone is known to be increased by CHT [27]. The results also showed that the presence of TDZ had a significant effect on the IAA levels of the tissues compared to those grown on a TDZ-free medium. Application of TDZ elicited effects typically associated with CKs. Studies have indicated that TDZ may act by modulating endogenous plant growth regulators [40]. In this regard, [41] reported that the application of TDZ in tissue culture medium increases internal CKs levels. There are also indicates the induction of accumulation or synthesis of endogenous CKs by TDZ [42]. Also, TDZ is involved in auxin synthesis by increasing levels of IAA and its precursor tryptophan and modification of cell membranes, energy levels, nutrient uptake, or nutrient assimilation [43]. There has been some evidence that TDZ may influence the endogenous content of the IAA. Peanut plants grown on a culture medium containing TDZ showed increased IAA, indicating that TDZ may enhance Aux synthesis [44]. A combination of CKs with auxins showed superior results for the shoot regeneration in date palm [34]. It has also been shown that the relative amounts of Auxs and CKs are essential for many physiological processes. The decrease in IAA levels at 15 mg L^−1^ CHT + 0.5 mg L^−1^ TDZ and increase in tissues treated with 20 mg L^−1^ CHT + 1.0 mg L^−1^ TDZ is consistent with the previous results putative mechanisms of shoot regeneration. The buds are regenerated when there is a high ratio of CKs to Auxs. Differences in regeneration abilities under different treatments may be due to differences in endogenous levels of growth hormones in these tissues. Several researchers have well documented the utility of molecular analysis of in vitro regenerated plantlets [35, 45, 46]. Somaclonal variation is often induced by the culture media and subculture cycles [47]. Therefore, testing of genetic stability of in vitro raised plants is necessary to date palm plantlets production. The applicability of RAPD technology has captured the interest of many researchers. Perhaps the main reason for the success of RAPD analysis is the acquisition of many genetic markers that require small amounts of DNA without cloning, sequencing, or any other form of molecular genome characterization. RAPD analysis micropropagated plants date palm cv Barhee indicated a profile similar to that of the mother plant that clearly showed the genetic stability of those plants (Fig. 5a–d). This confirmed that the in vitro regenerated P*. dactylifera* L. cv Barhee was genetically stable and free of clonal variations. For induction of defense responses and stimulation of cell division, CHT may directly affect gene expression interacting with chromatin [48]. A successful in vitro propagation method should give true-to-type plantlets without the occurrence of genetic mutations or morphological alteration [49–51]. Similarly, no variation appeared in genetic variation using RAPD has been reported in many cases of in vitro propagated of chestnut rootstock and almond plantlets hybrids [52, 53]. Terminalia arjuna [54], and medicinal herb–Coleus aromaticus Benth L. [55] *P. dactylifera* L. cv Ashgar [35]. On the other hand, cytokinins play a major role in DNA synthesis, cell division, and plant regeneration and regulate protein synthesis responsible for forming the mitotic spindle [56]. The RAPD technique showed genetic conformity of micro propagated plants of H. procumbent, pretreated or non-pretreated with TDZ [57] (Table 3).

**Table 3:** Effect of chitosan (CHT) and thidiazuron (TDZ) on a response percentage (%) of callus for bud formation, and a number of buds/100 mg callus for date palm, cv. Barhee after 12 weeks of culturing

Treatments (mgL^−1^)	frequency [%]	Shoot number	Treatments(mgL^−1^)	frequency [%]	Shoot number
**0.0 CHT + 0.0 TDZ**	0.0 ± 0.0	0.0 ± 0.0	**10 CHT + 1.0 TDZ**	41.67 ± 3.89	6.60 ± 0.58
**0.0 CHT + 0.5 TDZ**	25.0 ± 4.81	3.0 ± 0.50	**15 CHT + 0.0 TDZ**	25.0 ± 4.81	3.33 ± 0.4
**0.0 CHT + 1.0 TDZ**	16.67 ± 1.53	2.5 ± 0.22	**15 CHT + 0.5 TDZ**	75.00 ± 4.60	16.11 ± 0.99
**5 CHT + 0.0 TDZ**	8.34 ± 1.21	2.0 ± 0.19	**15 CHT + 1.0 TDZ**	58.34 ± 4.81	13.2 ± 0.59
**5 CHT + 0.5 TDZ**	41.67 ± 3.89	7.4 ± 0.58	**20 CHT + 0.0 TDZ**	25.0 ± 4.81	3.66 ± 0.42
**5 CHT + 1.0 TDZ**	33.34 ± 3.05	6.25 ± 0.4	**20 CHT + 0.5 TDZ**	58.34 ± 4.81	11.14 ± 0.17
**10 CHT + 0.0 TDZ**	16.67 ± 1.53	2.5 ± 0.22	**20 CHT + 1.0 TDZ**	41.67 ± 3.89	9.2 ± 0.21
**10 CHT + 0.5 TDZ**	58.34 ± 4.81	7.85±0.1			
**LSD < 0.05**	**13.9**	**0.7**		**13.9**	**0.7**

± Standard error (n12)

## Conclusions

To the best of our knowledge, this is the first study on the efficacy of CHT and TDZ together on genetically stable multiple shoot developments of date palm. This report provides an efficient protocol for a higher frequency of genetically stable multiple shoot development and a complete plant proliferation system through callus tissues. The combination of CHT and TDZ showed a positive effect on multiple shoot induction compared with either individual application or control treatment. Adding 15 mgL^−1^ CHT to the regeneration medium supplemented with 0.5 mgL^−1^ TDZ was the best combination to promote the growth of date palm cv Barhee as it resulted in the highest response rate and the number of shoots. CKs content increased in in vitro shoots regenerated in the same combination mentioned above. Random amplified polymorphic DNA of treatments with CHT and TDZ showed genetic similarity to mother plants. We conclude that applying the strategy of cultures treatment with CHT and TDZ may be valuable for improving date palm cv Barhee in vitro propagation. However, further studies are required to evaluate the effects of CHT.

## Data Availability

All data generated or analyzed during this study are included in this article.

## Abbreviations

NAA: Naphthaleneacetic acid
2iP: N6-(2-Isopentenyl) adenine
IAA: Indole acetic acid introduction
Aux: Auxins
CKs: Cytokines
CHT: Chitosan
TDZ: Thidiazuron
MS: Murashige and Skoog Basal Medium
RAPD profiles: Random amplified polymorphic DNA
